# Anti-inflammatory effects of curcumin are associated with down regulating microRNA-155 in LPS-treated macrophages and mice

**DOI:** 10.1080/13880209.2017.1297838

**Published:** 2017-03-06

**Authors:** Feiya Ma, Fei Liu, Liang Ding, Ming You, Huimin Yue, Yujie Zhou, Yayi Hou

**Affiliations:** a The State Key Laboratory of Pharmaceutical Biotechnology, Division of Immunology, Medical School, Nanjing University, Nanjing, PR China;; b The Affiliated Drum Tower Hospital of Nanjing University Medical School, Nanjing, PR China;; c Jiangsu Key Laboratory of Molecular Medicine, Medical School, Nanjing University, Nanjing, PR China

**Keywords:** Phosphoinositide 3-kinase (PI3K), AKT, inflammation, sepsis, p85α

## Abstract

**Context:** The natural polyphenolic compound curcumin has been proved to modulate innate immune responses and possess anti-inflammatory properties. Nevertheless, the mechanism remains poorly understood, particularly regarding curcumin-regulated miRNAs under inflammatory response.

**Objective:** This study investigates the role of miRNA-155 in the effects of curcumin on inflammatory response in cell and a mouse model.

**Materials and methods:** The anti-inflammatory activity of curcumin (5, 10 and 15 μM, 2 h) in lipopolysaccharide (LPS, 200 ng/mL)-induced cells were measured by quantitative PCR. The animals were treated orally by 20 mg/kg curcumin for 3 days before an LPS intraperitoneal injection (10 mg/kg, 16 h). MicroRNA (miRNA) expression and the underlying molecular mechanisms were assessed using transfection technique and western blotting.

**Results and discussion:** Curcumin efficiently inhibited LPS-induced cytokines (TNF-α, IL-6) and microRNA-155 (miR-155) expression (*p* < 0.05) without affecting the normally growth of Raw264.7 and THP-1 cells (IC_50_ 21.8 and 22.3 μM at 48 h, respectively). Moreover, the levels of cytokines were suppressed by curcumin in miR-155 mimics transfected cells (*p* < 0.05). A blockade of PI3K/AKT signalling pathways resulted in a decreased level of miR-155 (*p* < 0.05). Curcumin effectively protected mice from sepsis as evidenced by decreasing histological damage, reducing AST (352.0 vs 279.3 U/L), BUN (14.8 vs 10.8 mmol/L) levels and the proportion of macrophages in spleen (31.1% vs 13.5%). MicroRNA-155 level and cytokines were also reduced in curcumin-treated mice (*p* < 0.05).

**Conclusions:** Curcumin’s ability to suppress LPS-induced inflammatory response may be due to the inhibition of miR-155.

## Introduction

The innate immune system is activated to release pro-inflammatory cytokines such as IL-6 and TNF-α during infections and injuries. These cytokines then initiate the development of inflammatory responses and play an important role in controlling infection (Basnet & Skalko-Basnet 2011). Lipopolysaccharide (LPS), the product of Gram-negative bacteria, is an important inflammatory factor and has been widely used to induce various inflammation models *in vitro* and *in vivo* when studying inflammation-related diseases (Bascands et al. 2009; Cabrera-Benitez et al. 2012).

Curcumin is a natural polyphenol derived from the rhizomes of the plant *Curcuma longa* L. (Zingiberaceae) and commonly used as a spice and food-colouring material. It also has long been used as a naturally occurring medicine to treat various diseases associate with inflammation. In recent years, curcumin has been reported to reduce the inflammatory response by regulating the production of inflammatory molecules *in vitro* models (Srivastava et al. 2011; Gupta et al. 2012). Studies have shown that curcumin can relieve the IMQ-induced psoriasis-like inflammation in a mouse model by impacting the IL-23/IL-17 A axis and then down-regulating IL-17 A/IL-22 production indirectly (Sun et al. 2013). Furthermore, curcumin treatment exerted a significant anti-inflammatory effect on *Helicobacter pylori* infected mucosal disease, pointing to the promising role of a nutritional approach in the prevention of *H. pylori-*induced deleterious inflammation (Santos et al. 2015). As numerous articles reported, curcumin promoted the degradation of TNF-α and IL-6 expressed by LPS stimulation (Zhu et al. 2016). And the phosphoinositide 3-kinase (PI3K)-AKT signalling pathway plays a crucial role in the anti-inflammatory effects of curcumin in LPS-activated inflammatory responses (Cianciulli et al. 2016). Various studies also provide strong evidence for the beneficial effects of curcumin in various inflammation-related diseases including sepsis (Vachharajani et al. 2010).

MicroRNAs (miRNAs) are small noncoding RNAs that regulate eukaryotic gene expression by direct sequence-specific targeting 3′-untranslated regions (UTR) of mRNA, which result in translational repression or mRNA degradation (Huntzinger & Izaurralde 2011). MiRNAs have been implicated in the pathogenesis of common human diseases, such as neurologic, cardiovascular, cancer, inflammatory and autoimmune diseases (Iborra et al. 2012). Moreover, miRNAs regulate autoimmunity and inflammation by affecting the differentiation, maturation, and function of various immune cells (Xu & Zhang 2016). Specific miRNAs such as miR-146, miR-132 and miR-155 can be activated by inflammatory mediators and microbial components (Taganov et al. 2006). Among these miRNAs, miRNA-155 (miR-155) in particular has emerged as a key transcriptional regulator of some inflammation-related diseases (Tili et al. 2015). On the cellular level, the expression of miR-155 is strongly induced by different Toll-like receptor (TLR) ligands including TLR4 ligand (LPS). Furthermore, miR-155 has been proposed in the regulation of immune functions and inflammatory processes in macrophages (Taganov et al. 2006; O'Connell et al. 2007). Numerous studies have shown that miRNAs are involved in the modulation function of curcumin on the inhibition of cancer cell growth (Gao et al. 2012; Subramaniam et al. 2012). Nevertheless, there is no report on the links between the function of curcumin and miRNAs in regard to inflammation currently.

This study investigates the effect of curcumin on LPS-induced miR-155 expression in cell lines and animals, and the involvement of miR-155 in the anti-inflammation process. Besides, we also observed the role of PI3K/AKT signalling pathway in this process.

## Materials and methods

### Chemicals and reagents

Curcumin (purity >98%, molecular formula: C_21_H_20_O_6_, molecular weight: 368.37) was purchased from Sigma, lipopolysaccharide (LPS, Escherichia coli 0111: B4, Sigma). Dulbecco’s modified Eagle’s medium (DMEM), penicillin G, streptomycin and foetal bovine serum (FBS) were purchased from Gibco Inc. (Grand Island, NY). Phorbol 12-myristate 13-acetate (PMA) was from Sigma.

### Cell culture

RAW264.7 cells (murine macrophage cell line, ATCC, Manassas, VA) were cultured in endotoxin-free DMEM supplemented with 10% FBS, 100 U/mL penicillin and 100 μg/mL streptomycin. THP-1 cells (human acute monocyte leukaemia cell line, ATCC) were cultured in RPMI-1640 supplemented with 10% foetal bovine serum plus antibiotics. THP-1 cells were differentiated with phorbol 12-myristate 13-acetate (PMA) at 20 ng/mL for 24 h. We removed the PMA containing media and incubated PMA-treated cells in RPMI-1640 for 24 h. Cells were incubated at 37 °C in a humidified atmosphere containing 5% CO_2_.

### *In vitro* toxicity assay

Cell viability was performed to detect the effect of curcumin by a CCK-8 Kit (DojinDo Molecular Technologies, Gaithersburg, MD) in accordance with the manufacturer’s instructions. RAW264.7 cells or human THP-1 cells were seeded in 96-well plates at a density of 2 × 10^4^ cells/well, treated with curcumin in different concentrations (2–20 μM) for 24 h and 48 h. Then CCK-8 (10 μL) was separately added to each well and cultured for another 2 h. OD absorbance was measured at 450 nm using a multi-detection micro plate reader (Bio-Tek, Winooski, VT).

### Detection of miRNA and mRNA expression

Gene expression was determined by quantitative real-time PCR (qPCR). Total RNA including miRNAs was extracted using Trizol reagent (Invitrogen, Carlsbad, CA) and was dissolved in RNase-free water according to the manufacturer’s instructions. Total RNA (1 μg) was used as a template for single strand cDNA synthesis, using SYBR Green PCR Master Mix for RT-qPCR on an ABI Vii 7 detection system (Applied Biosystems Inc., Foster City, CA). And the relative expression calculated using the 2^−ΔΔ^CT method was performed to analyze the results, with GAPDH as an internal control. The method to quantify miRNAs was performed by stem-loop RT-PCR. MiRNAs and reverse primers were put at 65 °C for 5 min to form highly target-specific stem-loop structure. Then reverse transcriptase, RNase inhibitor, dNTPs and buffer were added for reverse transcription. MiRNAs amplification was also performed by using an ABI Vii 7 detection system with SYBR Green dye (Invitrogen, Carlsbad, CA), quantification data were presented as a ratio to the U6 level. Primer oligonucleotides were synthesized by Invitrogen. The sequences of the primers are listed in Table 1. All experiments were performed in triplicate (*n* = 3).

**Table 1.** Primers used for real-time quantitative PCR analysis.

Gene	Sequence (5′to 3′)
m-GAPDH-F	AACGACCCCTTCATTGAC
m-GAPDH-R	TCCACGACATACTCAGCAC
m-TNF-α-F	CCCTCACACTCAGATCATCATCTTCT
m-TNF-α-R	GCTACGACGTGGGCTACAG
m-IL-6-F	TAGTCCTTCCTACCCCAATTTCC
m-IL-6-R	TTGGTCCTTAGCCACTCCTTC
m-miR-155-5p-F	ACACTCCAGCTGGGTTAATGCTAATTGTG
m-miR-155-5p-R	CTCAACTGGTGTCGTGGAGTCGGCAATTCAGTTGAGACCCCTAT
m-socs1-FP	CTG CGG CTT CTA TTG GGG AC
m-socs1-RP	AAA AGG CAG TCG AAG GTC TCG
h-GAPDH-F	AGAAGGCTGGGGCTCATTTG
h-GAPDH-R	AGGGGCCATCCACAGTCTTC
h-miR-155-5p-F	ACACTCCAGCTGGGTTAATGCTAATCGTGAT
h-miR-155-5p-R	CTCAACTGGTGTCGTGGAGTCGGCAATTCAGTTGAGACCCCTAT
h-IL-6-F	TTCGGCAAATGTAGCATG
h-IL-6-R	AATAGTGTCCTAACGCTCATAC
h-TNF-α-F	CCTCTCTCTAATCAGCCCTCTG
h-TNF-α-R	GAGGACCTGGGAGTAGATGAG
has-Bic-F	CTCTAATGGTGGCACAAA
has-Bic-F	TGATAAAAACAAACATGGGCTTGAC
mmu-Bic-F	GACACAAGGCCTGTTACTAGCAC
mmu-Bic-R	GTCTGACATCTACGTTCATCCAGC

### MiRNA transfection

Over expression of miR-155 in cell line was achieved by transfecting cells with miR-155 mimic (double-stranded chemically modified RNA oligonucleotides, Gene Pharma Shanghai, China). Cells were seeded in 12-well plates at a concentration of 1 × 10^5/^mL and cultured in medium without antibiotics for ∼24 h before transfection. Then cells were transiently transfected with a miR-155 mimics or a negative control (NC) at a final concentration of 50 nM. After transfection, the medium was replaced with fresh incubation medium contained FBS after 6 h incubation at 37 °C and 5% CO_2_. Cells were harvested and subjected to other treatments after transfection, and miR-155 negative control was transfected as matched controls. Transfections are performed with lipofectimine 2000 reagents (Invitrogen, Carlsbad, CA).

### Western blot analysis

Protein extracts using lysis buffer and protease inhibitor (Roche), then stored at −80 °C until use. Each respective protein (50 μg) was boiled in SDS-PAGE loading buffer, subjected to gel electrophoresis with Tris-glycine running buffer and then electrophoretically transferred onto PVDF membranes. After blocking with 5% BSA in 0.1% Tween 20 containing TBS, membranes were incubated with corresponding primary antibodies against GAPDH, AKT, ERK1/2, p38 MAPK, c-Jun N-terminal kinase1/2 (JNK1/2), phospho-AKT (Ser473), phospho-ERK1/2, phospho-p38, phospho-JNK1/2 (Cell Signaling Technology, Danvers, MA, all dilutions at 1:1000), phospho-PI3K p85α (Bioworld, Nanjing, China, dilutions at 1:1000), PI3K p85α (Proteintech, Wuhan, China, dilutions at 1:1000) followed by incubation with secondary antibodies horseradish peroxidase-conjugated anti-rat IgG (Beyotime, Jiangsu, China, dilution at 1:3000) using Plus Western blot detection reagents (Millipore, CA). The densities of bands were scanned and quantified using Image J software (Softonic, Barcelona, Spain).

### Sepsis mice

This study was carried out in accordance with institutional guidelines for the care and use of animals based on the Guide produced by Nanjing University’s Animal Care Commission; 6–8 weeks old male C57BL/6 mice weighing 21 ± 2 g were obtained from Model Animal Research Center at Nanjing University. All mice were maintained under specific pathogen-free conditions and 12 h light/dark cycles. In the study’s animal experiments, the sepsis model was built by intraperitoneal injection of LPS (10 mg/kg). C57BL/6 mice were divided into three groups of 6 animals randomly as follows: Group a: carboxymethylcellulose solution; Group b: LPS (10 mg/kg); Group c: LPS + Cur (20 mg/kg). Mice were pretreated (3 days) with curcumin in 0.5% carboxymethylcellulose (Sigma-Aldrich) via oral gavage before an LPS intraperitoneal injection and then sacrificed at 16 h.

### Determining biochemical parameters in serum

Blood samples were obtained from mouse. The levels of serum aspartate transaminase (AST) and blood urea nitrogen (BUN) were determined using an automatic biochemical analyzer Beckman Coulter AU5800 (Beckman Coulter, CA).

### Flow cytometric analysis

Anti-mouse F4/80-PE, CD11c-FITC antibodies were purchased from eBioscience (San Diego, CA). Flow cytometry was performed with a FACSCalibur flow cytometer (BD, San Diego, CA), and the data were analyzed using Flow Jo (Treestar, Inc., San Carlo, CA).

### Statistical analysis

Results were expressed as mean ± SEM of three experiments. Significant differences in the mean values were evaluated by two-way ANOVA or Student’s *t*-test using Instat 2 (GraphPad, La Jolla, CA). Statistical significance was considered at *p* < 0.05.

## Results

### Curcumin inhibits the production of LPS-induced pro-inflammatory cytokines in RAW264.7 cells

**Figure 1.**
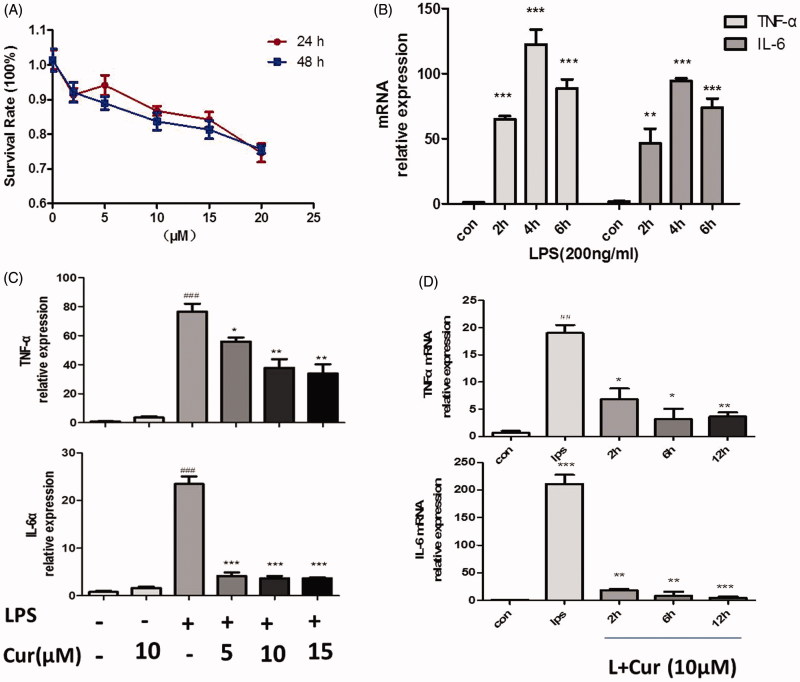
Curcumin inhibits LPS-induced production of pro-inflammatory mediators in macrophages RAW264.7 cells. (A) The effect of curcumin on cell viability assay. (B) TNF-α and IL-6 at the mRNA levels, cells were stimulated with LPS at different culture time (0, 2, 4, 6 h). (C) Cells were treated with or without curcumin (5, 10, 15 μM) for 2 h before LPS (200 ng/mL) stimulation for another 4 h. (D) Cells were treated with curcumin in different culture times (0, 2, 6, 12 h), then induced with LPS for 4 h. Expression levels of TNF-α and IL-6 mRNA were tested by real-time quantitative PCR (Q-PCR). The data are representative of at least three independent experiments (mean ± SEM) (##*p* < 0.01, ###*p* < 0.001 vs control; *p < 0.05; **p < 0.01, ***p < 0.001 vs LPS alone.).

In response to infection, macrophages are the most efficient pathogen scavengers and the predominant source of inflammatory cytokines (Schletter et al. 1995). Initially, we sought to determine suitable concentrations of curcumin on RAW264.7 cells, the viability of cells were examined by the CCK-8 kit assay. Results showed that when concentration of curcumin was lower than 15 μM. The survival rates at 24 and 48 h were greater than 84.2 ± 4.9% and 81.3 ± 5.7% (Figure 1(A)). The IC_50_ values were 31.22 and 21.87 μM, respectively. As such, it was determined that the study use 5, 10, and 15 μM as the experimental concentrations. Besides, according to our experiments, the minimum effective concentration is 3 μM (data not shown). Since stimulation of RAW264.7 with LPS caused a significant increase in IL-6 and TNF-α secretion and reached the maximal levels by around 4 h (Figure 1(B)), we exposed cells with different concentrations of curcumin for 2 h, then stimulated with LPS (200 ng/mL) for another 4 h. Data showed that curcumin remarkably decreased LPS-induced secretion of pro-inflammatory mediators in mouse macrophage at different concentrations (*p* < 0.05) (Figure 1(C)). However, treating cells with curcumin alone had no effect on the expression of cytokines. Moreover, curcumin decreased the secretion of TNF-α and IL-6 at different times as can be seen in Figure 1(D) (*p* < 0.05). These results indicated that the production of pro-inflammatory mediators was clearly reduced by curcumin at various time-points and concentrations.

### The inhibitory effect of curcumin on pro-inflammatory cytokines are related to miR-155

**Figure 2.**
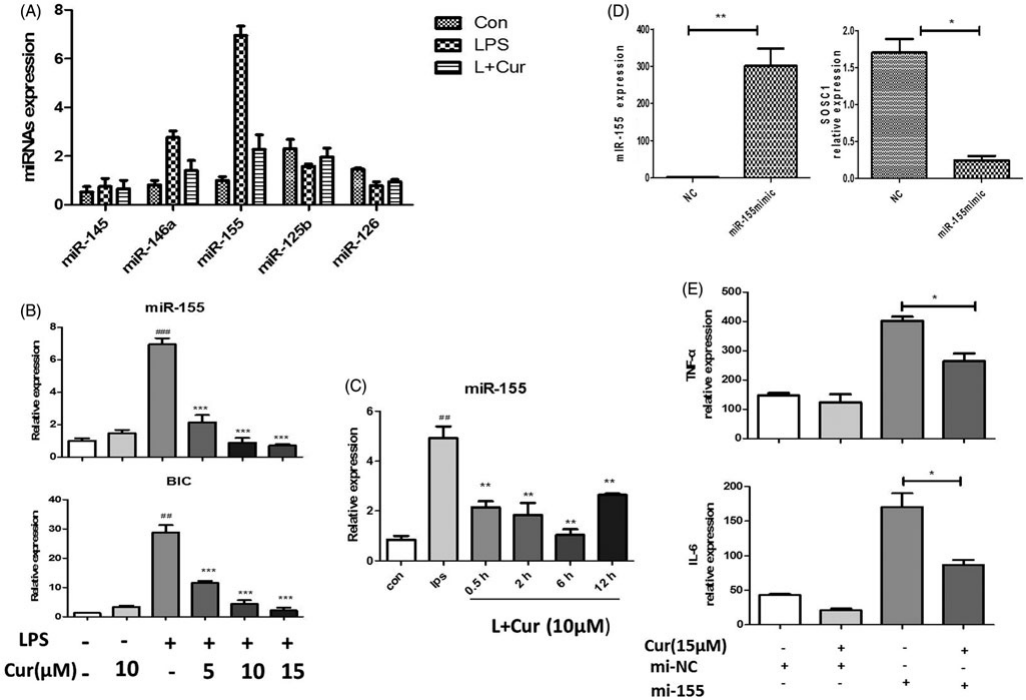
Inhibitory effect of curcumin on pro-inflammatory expression is related to miR-155 in RAW264.7 cells. (A) The expression of different miRNAs were measured by real-time quantitative-PCR (LPS 200 ng/mL, Cur 10 μM). (B) MiR-155 and BIC levels. Cells treated with or without curcumin in different concentrations (5, 10, 15 μM) under stimulation of LPS. (C) MiR-155 expression was detected in the cells that treated with curcumin and LPS in different culture time. (D) Cells were transfected with miR-155 mimics (mi-155) or negative control mimics (mi-NC) (50 nM) for 48 h, miR-155 and SOCS1 expression were assayed by Q-PCR. (E) mRNA level of cytokines in miR-155 or negative control mimics transfected cells with the treatment of curcumin (15 μM). The data are presented as the mean ± SEM of at least three independent experiments (##*p* < 0.01, ###*p* < 0.001; *p < 0.05, **p < 0.01, ***p < 0.001).

Subsequently, in order to investigate how curcumin regulated cytokines, we measured the expression of several miRNAs associated with inflammation by using a Q-PCR based approach. Among these miRNAs, miR-155 showed the most obvious change. Stimulating of RAW264.7 cells with LPS resulted in a seven-fold increase in miR-155 (Figure 2(A)). And miR-155 has been reported as a key transcriptional regulator of some inflammation-related diseases in numerous studies. Pre-treatment using increasing concentrations of curcumin significantly decreased LPS-induced miR-155 expression (Figure 2(B)). Simultaneously, results showed that curcumin could inhibit LPS-induced pri-miR-155 (BIC) as well (Figure 2(B)), demonstrating that miR-155 is regulated by curcumin at the transcriptional level. Moreover, curcumin also exhibited an inhibitory effect on miR-155 at various time-points (Figure 2(C)). In an attempt to determine whether miR-155 is a target of curcumin, the study had RAW264.7 cells transfected with miR-155 dsRNA mimic (miR-155 mimic). These transfected cells showed much higher levels of miR-155 compared with that of the negative mimic transfected cells (Figure 2(D)). Suppressor of cytokine signaling-1 (SOCS1), a well-known target of miR-155 (Kinjyo et al. 2002; Nakagawa et al. 2002), was declined after been transfected with miR-155 mimics (Figure 2(D)). These results indicated that the transfection of miR-155 mimics was successful. Furthermore, we observed the increased level of TNF-α and IL-6 in miR-155 mimics transfected cells. However, transfected cells that later added curcumin in the miR-155-abundant cells showed clear reduced production of TNF-α and IL-6 (Figure 2(E)). This demonstrated that curcumin could repress the inflammatory responses by targeting miR-155. Thus, these results suggested miR-155 plays a vital role in inhibiting LPS-induced cytokine production in curcumin-treated mouse macrophages.

### Curcumin inhibits inflammatory functions through miR-155 in human THP-1 cells

**Figure 3.**
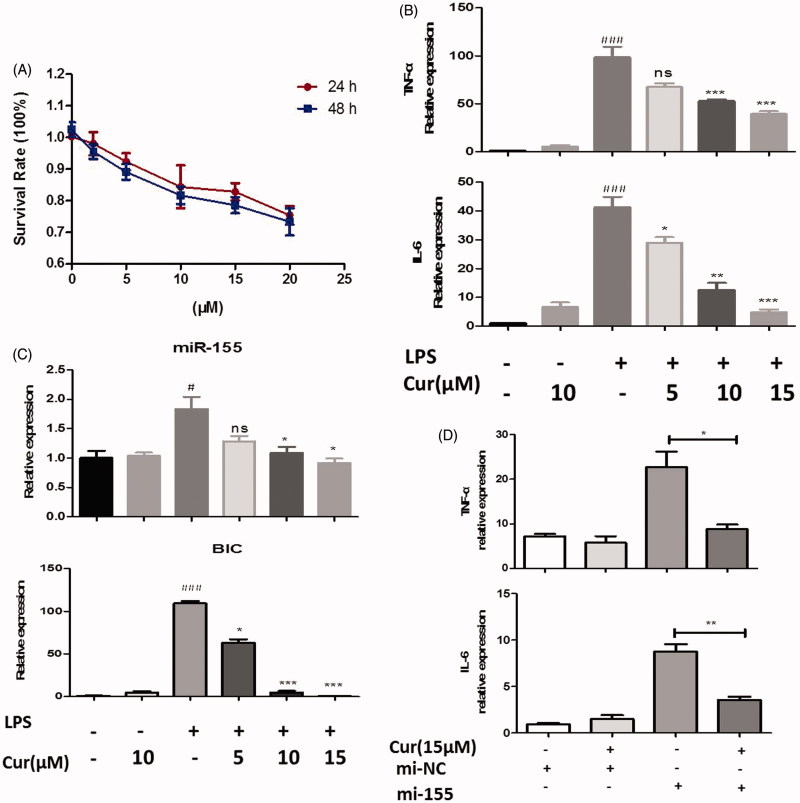
Curcumin suppresses the level of miR-155 in human THP-1 cells. (A) The effect of curcumin on cell viability assay. (B,C) Cells were treated with or without curcumin (5, 10, 15 μM) for 2 h before LPS (200 ng/mL) stimulation for another 4 h, then mRNA levels of cytokines (B) and miR-155, BIC expression (C) were measured. (D) mRNA level of cytokines in miR-155 or negative control mimics transfected cells with the treatment of curcumin (15 μM). The data shown represent the mean values of three independent experiments, and the error bars represent the SEM. (#*p* < 0.05, ###*p* < 0.001 vs control; *p < 0.05, **p < 0.01,***p < 0.001 vs LPS alone; ns denotes *p* > 0.05).

To ensure that changes in miRNA expression were not restricted to the mouse RAW264.7 cell line, we also examined the responses in human monocyte THP-1 macrophage. The viability of THP-1 cells was also examined by the CCK-8 kit assay after treated with curcumin. When the concentration of curcumin was lower than 15 μM, the survival rates at 24 and 48 h were greater than 83.2 ± 6.3% and 80.2 ± 5.3% (Figure 3(A)). The IC_50_ values were 30.34 μM and 22.30 μM, respectively. Based on the results of cell viability mentioned above, concentrations of 5, 10, and 15 μM were used for each respective experiment. Cells were treated with different concentrations of curcumin for 2 h prior to LPS stimulation (200 ng/mL) for 4 h. We first examined the mRNA expression of cytokines: similarly, it revealed that pretreatment with curcumin clearly reduced mRNA expression of IL-6 and TNF-α (*p* < 0.05) (Figure 3(B)). Furthermore, the expression of miRNA-155 and BIC in THP-1 cell line also declined in curcumin pretreatment group as compared to the group given only LPS stimulations (*p* < 0.05) (Figure 3(C)). In addition, cells were transfected with miR-155 mimics before being treated with curcumin. TNF-α and IL-6 production was substantially enhanced in the miR-155 abundant cells when compared to negative mimic transfected cells (*p* < 0.05). However, treated with curcumin significantly reduced the production of TNF-α and IL-6 in the miR-155 abundant cells (Figure 3(D)), which demonstrated that curcumin could repress the inflammatory responses by targeting miR-155 in THP-1 cells.

### Curcumin lowers miR-155 levels through PI3K/AKT pathway

**Figure 4.**
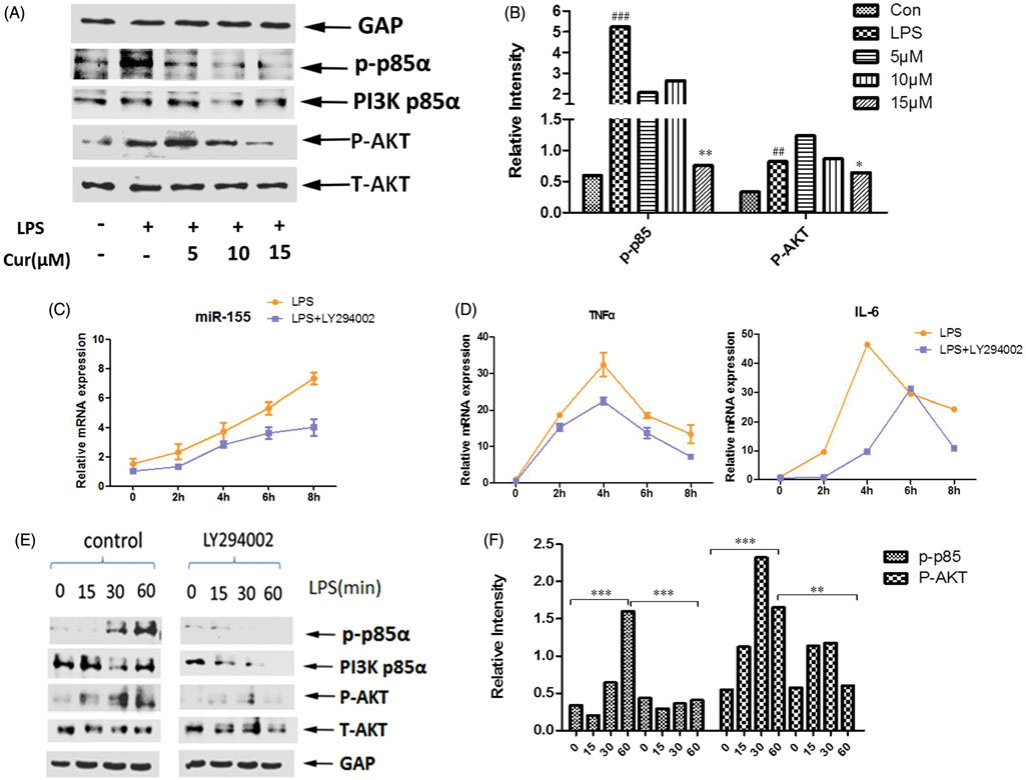
Curcumin inhibits miR-155, TNF-α and IL-6 through PI3K-AKT pathway. (A,B) Western blot analysis of PI3K p85α, p-p85α, AKT and the phosphorylation of AKT. Cells were pretreated with curcumin (5, 10, 15 μM) for 2 h, followed by stimulation with LPS for 30 min. Bands were quantified by Image J software. (C,D) Raw264.7 cells were pretreated with or without the inhibitor of PI3K (LY294002) for different time periods (0, 2, 4, 6, 8 h) before LPS stimulation for 4 h. MiR-155 (C) and mRNA levels of TNF-α and IL-6 (D) were assayed. (E,F) Western blot analysis of PI3K p85α, p-p85α, AKT, P-AKT. Raw264.7 cells were pretreated with or without LY294002 2 h followed by stimulation with LPS for 0, 15, 30, 60 min. The data are presented as the mean ± SEM of at least three independent experiments.

After determining these effects, the study turned to investigate how curcumin modulates the expression of miR-155. Reports have shown that surface activation of TLR4 invokes the phosphatidylinositol 3-kinase (PI3K)/AKT pathway (Ojaniemi et al. 2003; Laird et al. 2009). To detect whether PI3K/AKT pathway regulates miR-155 in its ability to inhibit cytokines, we analyzed PI3K activation and the phosphorylation of AKT in curcumin and LPS-treated Raw264.7 macrophages. Results from a Western blot analysis revealed that the phosphorylation of PI3K p85α and AKT levels decreased significantly when co-incubated with curcumin (5-15 μM) and LPS (Figure 4(A, B)). We deduced that these results may bring out the crucial role of PI3K in its regulation of miR-155.

For further study, cells were pretreated with the PI3K inhibitor (LY294002, Beyotime, Jiangsu, China) before LPS preparation. We measured miR-155 expression and the downstream synthesis and secretion of inflammatory cytokine response to LPS in PI3K inhibited cells, where production of miR-155 is reduced in response to LPS as expected, when compared to control cells (Figure 4 (C)). Similar results were obtained in pro-inflammatory cytokines, IL-6 and TNF-α (Figure 4(D)). At the protein level, quantification revealed an obvious decrease in the phosphorylation of AKT at each incubation time-point (15, 30, and 60 min, respectively) of LPS treatment, which is correlated to the depletion level of PI3K (Figure 4(E, F)). In conclusion, the PI3K/AKT pathway is required for curcumin’s inhibition of miR-155 in response to pathogen recognition by TLR4.

### Curcumin affects the phosphorylation of STAT-1 and MAPK signaling

**Figure 5.**
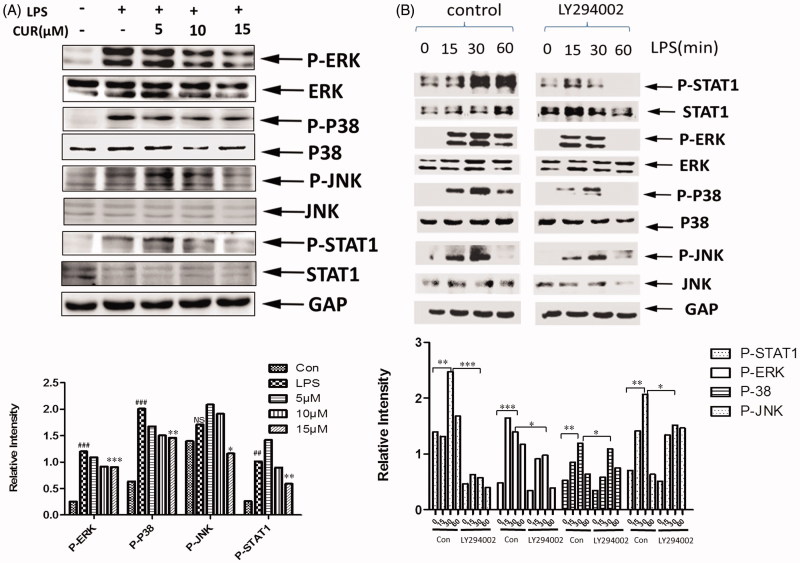
Curcumin inhibits the phosphorylation of STAT-1 and MAPK signaling (A) The levels of P-ERK, P-P38, P-JNK and P-STAT-1 in cells were measured by Western blot analysis. Bands were quantified by Image J software. (B) Western blot analysis of P-ERK, P-38, P-JNK, P-STAT-1. Cells were pretreated with or without LY294002 for 2 h followed by stimulation with LPS for 0, 15, 30, 60 min.

As an important regulator of the immune system, miR-155 has multiple targets, such as SOCS1 and SHIP1, which are involved in immune functions (Kinjyo et al. 2002; Nakagawa et al. 2002; Bandyopadhyay et al. 2014). The transducer and activator of transcription 1 (STAT-1), and mitogen-activated protein kinase (MAPK) signalling pathways have both been implicated as targets for regulation by these proteins in macrophages (Kinjyo et al. 2002; Baetz et al. 2004). Some articles have also reported the relationship between miR-155 and MAPK, STAT-1 (Martin et al. 2014; Lin et al. 2015). Interestingly, when measuring STAT-1 and MAP kinase, we found that the phosphorylation of STAT-1 and ERK, P-38, JNK was reduced in congruence with increased concentrations of curcumin (Figure 5(A)). Meanwhile, the treatment led to the attenuation of LPS-induced P-ERK, P-JNK, P-38 and P-STAT-1 at each incubation time-point (15, 30, and 60 min) with obvious differences in the PI3K-blocked cells (Figure 5(B)). Thus, our results inferred that the inhibition on miR-155 by curcumin could mediate the downstream phosphorylation of ERK, P-38, JNK and STAT-1, with a possibility of this being due to the activity of some targets of miR-155.

### The administration of curcumin protects mice against LPS-induced sepsis

**Figure 6.**
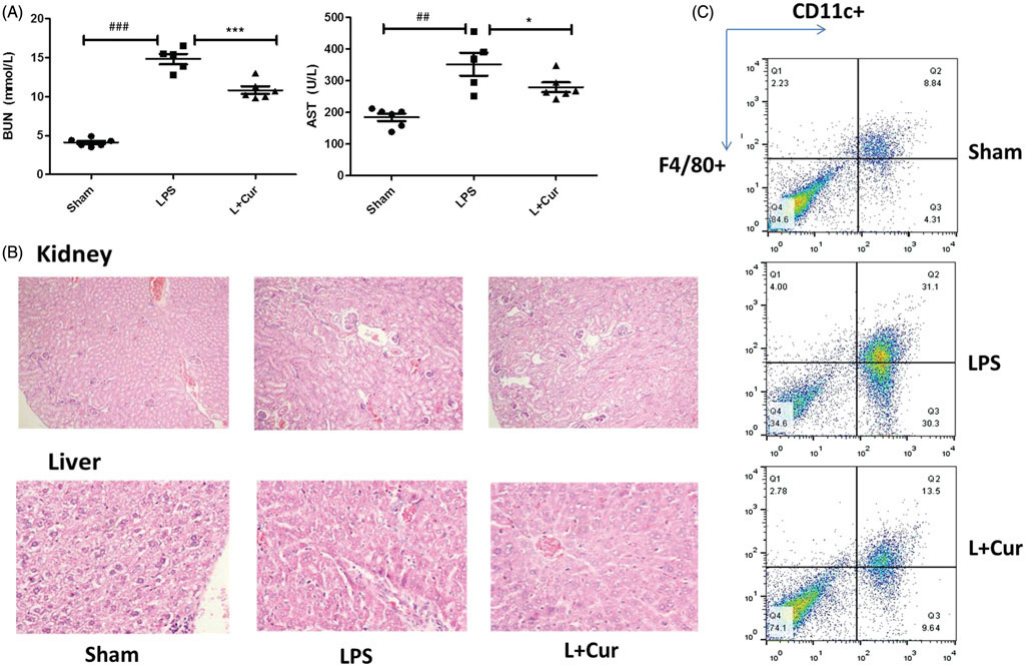
Curcumin ameliorates the histopathological damage in LPS-induced sepsis. Male C57BL/6 mice (*n* = 18) were randomly divided into three groups: a. carboxymethyl cellulose solution. b. LPS (10 mg/kg). c. LPS + Cur (20 mg/kg). (A) The levels of serum AST, BUN were determined using an automatic biochemical analyzer. (B) Hematoxylin and eosin staining of kidney and liver sections from mice assessed 16 h after LPS challenge. C. Representative proportions of macrophage (F4/80^+^CD11c^+^) in spleen from each group were detected using Flow Cytometry (##*p* < 0.01, ###*p* < 0.001 vs control; *p < 0.05, ***p < 0.001 vs LPS alone).

To extend our findings further, we sought to determine whether the use of curcumin would influence miR-155 in LPS-induced sepsis mice. In this study, functional changes in critical organs were observed. AST and BUN were known as the main function markers of liver and kidney, respectively. We detected the levels of serum AST and BUN and results indicated that these two parameters were higher in the LPS group (352.0 U/L, 14.8 mmol/L) compared to the sham group (184.3 U/L, 4.1 mmol/L), while in LPS + Cur group there was a significant drop (279.3 U/L, 10.8 mmol/L) (Figure 6(A)). Histologic assessment was performed after getting the tissues. Liver and kidney tissues (*n* = 6) from each experimental group were processed for histologic evaluation after HE staining (× 200). In the LPS group, significant damage was observed in the kidney and livers as evidenced by glomerular damage, severe dilation of the tubular lumen, moderate inflammatory cell infiltration, and vascular congestion. Treatment with curcumin (L + Cur) ameliorated these damages (Figure 6(B)). Results suggested that curcumin has protective effects on critical organs in mouse with sepsis.

**Figure 7.**
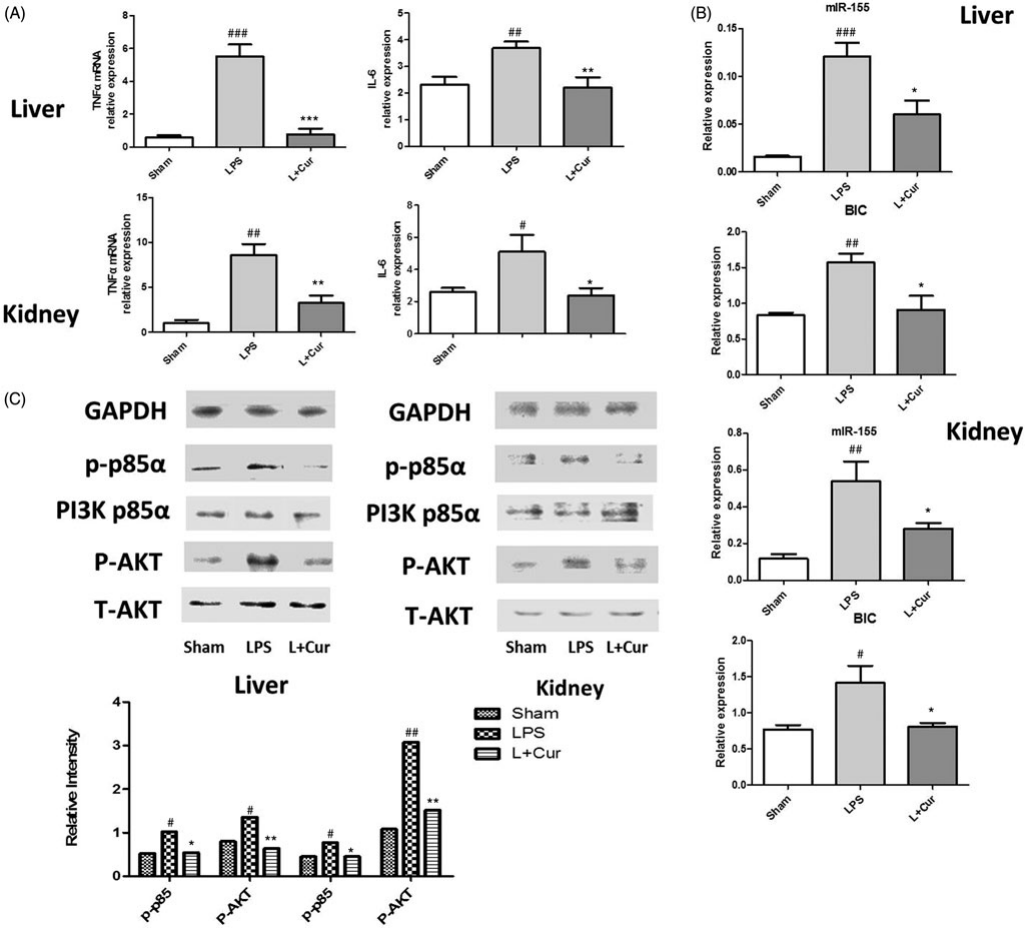
Curcumin suppresses the levels of miR-155 and BIC in sepsis mice. (A) mRNA levels of TNF-α and IL-6 in liver and kidney tissues were measured. (B) mRNA levels of miR-155 and BIC expression in tissues were measured by Q-PCR. (C) Western blot analysis of the levels of PI3K p85α, p-p85α, AKT and P-AKT, bands were quantified using Image J software. (#*p* < 0.05, ##*p* < 0.01, ###*p* < 0.001 vs control; *p < 0.05, **p < 0.01, ***p < 0.001 vs LPS alone).

The detection of F4/80^+^CD11c^+^ cells in mice spleen was performed with a FACSCalibur flow cytometer. We observed that LPS-induced group demonstrated significantly higher numbers of macrophages (31.1%) in comparison to the sham group (8.8%); however, it decreased with the treatment of curcumin (13.5%) (Figure 6(C)). Cytokines in both the liver and kidney tissues were enhanced in the LPS-induced sepsis group; following the introduction of curcumin, cytokines were found to be lower in curcumin-treated group (*p* < 0.05) (Figure 7(A)).

Most importantly, in order to investigate whether miR-155 and BIC were inhibited by curcumin in the LPS-induced sepsis model, we measured the levels of miR-155 and BIC expression. We found that in both the liver and kidney tissues, miR-155 and BIC revealed higher expression in the sepsis group. Nevertheless, curcumin was still able to reverse this situation (Figure 7(B)). Moreover, in sepsis mice, the protein level of p-p85α and the phosphorylation of AKT reduced obviously in the LPS + Cur group in comparison to the LPS group (Figure 7(C)). These results provide reference for our *in vitro* experiments very well. As such, our results demonstrated that curcumin protects mice from LPS-induced sepsis, and this process is closely related to the down-regulation of miR-155 expression.

## Discussion

The elaborate program was aimed at discovering the mechanism of antibacterial-induced inflammatory reaction by curcumin. The study utilized curcumin (a natural product known for its variety of attractive biological properties) to determine a series of reports on anti-inflammatory effects (Esatbeyoglu et al. 2012) and LPS, a well-known potent stimulant of the immune response system that is derived from Gram-negative bacteria (Rossol et al. 2011), which eventually leads to expression of many genes related to innate immunity and inflammation, and other gene products (Fan et al. 2001; Ma et al. 2005).

In the present study, curcumin was found to have an ability to inhibit the expression of IL-6 and TNF-α in mouse macrophage cell lines when stimulated with LPS. MiR-155 has also been identified as a positive regulator of the LPS signalling pathway, and as a common target for a broad range of inflammatory mediators through the PI3K/AKT pathway in monocytes and macrophages (Rajaram et al. 2011). Target analysis of miRNA expression using real-time PCR revealed that curcumin can down-regulate the expression of pro-oncogenic miR-20a, miR-17-5p, miR-21 and miR-27a in human tumor cell lines (Mudduluru et al. 2011; Gandhy et al. 2012), while the connection between curcumin and miRNAs in inflammatory responses is still unknown. Based on the functional changes and previous studies mentioned above, this study helped identify curcumin’s regulatory mechanism of the progress of inflammatory through miR-155. The data produced in this study concluded that LPS instigated the expression of miR-155, but using in combination with a pretreatment of curcumin would significantly decrease the expression of miR-155 in relation to increases in dosage. Moreover, an over-expression of miR-155 greatly reduced curcumin’s ability to inhibit LPS-induced IL-6 and TNF-α production. It is reported that when pri-155 promoter constructs fused to a luciferase reporter were transfected into Raw264.7 cells and treated with LPS (50–150 ng), the pri-155 promoter grows 5-fold at 100 ng, indicating that miR-155 is regulated by LPS at the transcriptional level (Quinn et al. 2014). Our study focused on the question of whether this induction was also inhibited by curcumin at the transcriptional level. Our results verified that pri-miR-155 induced by LPS is suppressed by curcumin. The above data together thus indicated that miR-155 holds a critical role in the regulation of curcumin’s macrophage inflammatory response.

PI3K is activated in response to LPS stimulation and has been noted in previous studies as an important factor in the signalling cascades triggered by LPS (Ojaniemi et al. 2003; Wang et al. 2014). The inhibition of PI3K results in an enhanced activation of LPS-induced MAPK activation and gene transcription (Guha & Mackman 2002). In our study, curcumin decreased the phosphorylation of p85α and AKT expression. More importantly, in the absence of PI3K, miR-155 levels exhibited clear reduction in size. Similar trends could be seen in AKT phosphorylation and downstream signalling. These findings revealed that PI3K plays a key part in the function of inhibiting miR-155 by curcumin and its presence controls a variety of downstream targets via PI3K/AKT pathway.

According to many previous studies, the interaction between miR-155 and its targets require a variety of immune cell types in order to achieve proper immunological functions (Huffaker & O'Connell 2015; Yang & Yang 2015). Our previous study found that, in both humans and mice, miR-155 directly targeted the 3′-untranslated region of CD1d upon TLR9 activation (Liu et al. 2015). SHIP1 and SOCS1 are also well-known targets of miR-155 and negatively regulate LPS-induced inflammation via different signaling (Kinjyo et al. 2002; Nakagawa et al. 2002; Bandyopadhyay et al. 2014). SOCS1 is a general regulator of cell signalling and regulates many cytokines, such as IL-2, IL-4, IL-6, IFN-γ, growth hormone, and erythropoietin, thus modulating various biological functions of immune cells (Alexander & Hilton 2004). The mRNA of PU.1, which is required for a late differentiation of B cells, was also identified as a possible target for miR-155 (James et al. 2004).

STAT-1, MAP kinase signalling pathways have been implicated as targets for regulation by SOCS1, SHIP1 and PU.1 in macrophages (Kinjyo et al. 2002; Baetz et al. 2004). We found that curcumin could affect both MAPK and STAT-1 dependent signal pathways, blocking PI3K activity could also lead to the decrease of MAPK and STAT-1 when stimulated with LPS. Based on the study’s results, it is possible that curcumin regulates miR-155 through the PI3K/AKT signalling pathway. Furthermore, miR-155 mediates the downstream phosphorylation of MAPK and STAT1 via targeted proteins such as SOCS1, PU1 and SHIP1. In this manner, LPS-induced immunological reactions can be reduced. Thus, we deduce that this anti-inflammatory mechanism is mediated by the down-regulation of miR-155, which may in turn affects SOCS1 and other targets. This influence thus allows both SOCS1 and other targets to increase their negative feedback regulatory mechanism.

Several articles have previously reported the inhibitory effect of curcumin on sepsis mice (Vachharajani et al. 2010; Zhang et al. 2014). We verified its anti-inflammatory effects *in vivo*. Kidney and liver are the two target organs most susceptible to sepsis. In other words, both liver and kidney functions could affect the development and prognosis of sepsis (Mårtensson & Bellomo 2015; Uddin et al. 2015). We found that curcumin play a protective role in LPS-induced sepsis model as evidenced by inflammatory markers and histopathologic examination. Most importantly, the levels of BIC and miRNA-155 in tissues declined in the curcumin pretreated group as compare to the LPS stimulation group. This reinforced our previous experiments on cell lines. Using a western blot analysis, we deduced that miR-155 play an anti-inflammatory role in sepsis through the PI3K/AKT pathway both in kidney and liver tissues. One interesting mechanism to be noted is that miR-155 is inhibited via the PI3K/AKT pathway, which would be a possible new strategy for the treatment of sepsis and other inflammatory disorders in the future. Finally, the specific role of miR-155 in immunological reactions yet to be fully understood, and more studies should be conducted to better understand it.

## Conclusions

This paper has demonstrated that curcumin’s inhibitory role in LPS-induced inflammatory responses is operated through mediating miR-155 and that inflammation therapies may be accomplished by blocking miR-155 at the transcriptional level. Furthermore, the reduced production of miR-155 in the PI3K-inhibited cells revealed that PI3K/AKT signalling is required for the inhibition of miR-155 in response to lipopolysaccharides.
